# Nanostructured Metal Oxide-Based Electrochemical Biosensors in Medical Diagnosis

**DOI:** 10.3390/bios14050238

**Published:** 2024-05-09

**Authors:** Gulsu Keles, Elif Sifa Ataman, Sueda Betul Taskin, İlker Polatoglu, Sevinc Kurbanoglu

**Affiliations:** 1 Department of Analytical Chemistry, Faculty of Pharmacy, Ankara University, 06560 Ankara, Türkiye; kelesgulsu@gmail.com; 2 Bioengineering Department, Manisa Celal Bayar University, 45140 Manisa, Türkiye; elifataman162@gmail.com (E.S.A.); suedabtl@gmail.com (S.B.T.)

**Keywords:** nanostructures, metal oxide nanomaterials, electrochemistry, biosensor, medical diagnosis

## Abstract

Nanostructured metal oxides (NMOs) provide electrical properties such as high surface-to-volume ratio, reaction activity, and good adsorption strength. Furthermore, they serve as a conductive substrate for the immobilization of biomolecules, exhibiting notable biological activity. Capitalizing on these characteristics, they find utility in the development of various electrochemical biosensing devices, elevating the sensitivity and selectivity of such diagnostic platforms. In this review, different types of NMOs, including zinc oxide (ZnO), titanium dioxide (TiO_2_), iron (II, III) oxide (Fe_3_O_4_), nickel oxide (NiO), and copper oxide (CuO); their synthesis methods; and how they can be integrated into biosensors used for medical diagnosis are examined. It also includes a detailed table for the last 10 years covering the morphologies, analysis techniques, analytes, and analytical performances of electrochemical biosensors developed for medical diagnosis.

## 1. Introduction

Nanomaterials comprise the most well-known category of materials, nanoparticles, as well as nano (i) ribbons, (ii) films, (iii) fibers, (iv) liquids, (v) spheres, (vi) tubes, (vii) rods, and (viii) wires; quantum dots; and hollow spheres. Numerous nanomaterials can be categorized based on their size, morphological structure, and other characteristics. These include carbon-based materials, semiconductors, polymers, lipid-based materials, and nanostructured metal oxides (NMOs) [1,2]. Attributable to their exceptional physical and chemical characteristics, like superparamagnetic behavior, cold welding properties, unique catalytic activity, sensitivity, selectivity, high stability, highly ionic nature, unusual adsorptive properties, fast diffusivities, lower melting points, no swelling variations, easy functionalization, simple modification to the desired size, porosity, shape, and easy incorporation into both hydrophobic and hydrophilic systems, NMOs are among the most widely used nanomaterials. They possess a high surface-to-volume ratio, adjusted surface working function, augmented surface reaction activity, potent catalytic effectiveness, and commendable adsorption capacity. These properties can be modified and controlled depending on the synthesizing methods of NMOs [3,4,5,6].

**Scheme 1:**
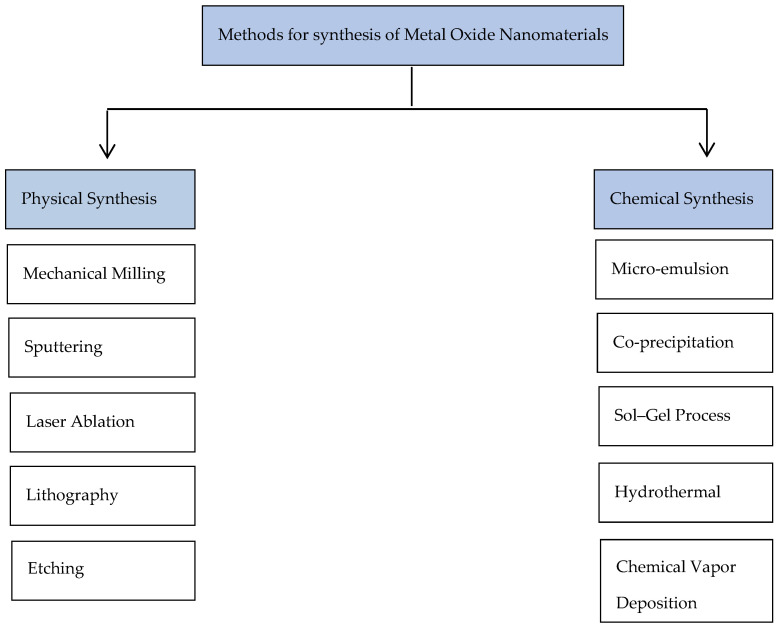
Some of the physical and chemical techniques used for the synthesis of NMOs [14,15].

There are many methods for synthesizing metal oxide, and they consist of two main groups: physical and chemical synthesis (Scheme 1) [7,8]. Some physical synthesis methods are mechanical milling, laser ablation, sputtering, lithography, and etching [9,10]. These techniques lack the ability to control particle sizes and structure, as they operate under a top-down methodology. This approach involves the disintegration of the bulk substance into smaller molecules, which subsequently undergo a conversion process to form nanoparticles. The most favored method is mechanical milling, wherein combinations of elemental or pre-alloyed powders undergo grinding inside specialized equipment capable of generating high-energy compressive impact pressures.

Examples of such equipment include attrition or shaker mills, and the process is conducted within a safeguarded and controlled environment [7,8,11,12,13]. The second method, chemical synthesis, includes the sol–gel operation, chemical vapor deposition, micro-emulsion, co-precipitation, and hydrothermal synthesis [9,10]. These approaches, grounded in a bottom-up methodology, are characterized by their simplicity, manageability, and efficacy. They facilitate precise control over nanoparticle size, composition, and morphology [11,12,13]. Crucial parameters influencing chemical synthesis include reducing agents, capping agents, and optimal temperature and pressure conditions. The sol–gel process has emerged as the favored technique, commencing with the formulation of a precursor mixture (sol or solution) that transitions into a more solid state through the solvent’s evaporation. Subsequently, desiccation and chemical bonding transpire among the solid particles or dissolved precursor substances [7,9,10].

**Figure 1:**
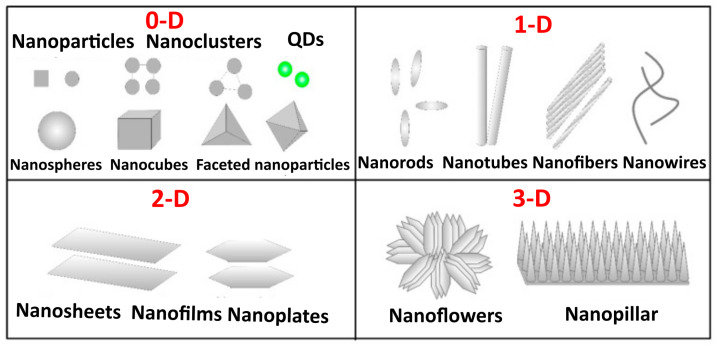
Scheme of zero-, one-, two-, and three-dimensional nanostructured materials with varied morphologies [6].

NMOs are classified into four groups based on their dimensions: zero-dimensional (0-D), one-dimensional (1-D), two-dimensional (2-D), and three-dimensional (3-D), as depicted in Figure 1 [6,16,17]. Small particles/clusters with three dimensions restricted to the nanoscale (1–100 nm) are called 0-D nanostructures [18]. Nanostructures with 0-D characteristics demonstrate a greater number of active edge sites, attributed to their intrinsic structural features such as elevated surface-to-volume ratios and extremely small sizes [19]. Zero-dimensional NMOs comprise fundamental elements, specifically nanoparticles, quantum dots, nanoclusters, and other materials whose all dimensions are nanometer-scale [6,17]. One-dimensional NMOs are widely acknowledged as suitable systems for investigating the size and dimensionality dependence of functional characteristics. They serve as effective platforms for exploring a broad spectrum of distinctive phenomena at the nanoscale [17]. In 1-D nanostructures, one dimension is outside the nanoscale, consisting of nanorods, nanotubes, nanowires, and nanofibers with linear geometric shapes [6,16]. Nanoplates, nanosheets, nanocoatings, and nanofilms, which feature two dimensions that are not within the nanoscale, are classified as planar or 2-D nanomaterials. When a material’s size exceeds 100 nm in all three orthogonal directions, 3-D nanomaterials are formed. This group includes nanopillars, nanoflowers, nanowires, multi-nanolayers, dendrimers, or bundles of nanotubes [6].

Metal oxide nanoparticles, nanofibers, nanocages, nanobelts, nanorods, nanotubes, and so on are commonly used as enhancement materials for electrochemical biosensors. The incorporation of nanoparticles into electrodes proves advantageous in enhancing sensor efficiency, attributable to their inherent properties [20]. They are used for biosensing applications, non-invasive sensing, drug delivery, in vitro and in vivo intracellular imaging investigations, and tissue engineering endeavors [21,22].

### 1.1. Nanostructured Metal Oxide-Based Biosensors

In recent years, biological sensors have been studied more extensively. Far-reaching benefits are provided by NMOs involved in biosensing research. In the context of the sensory utilization of NMOs, comprehensive analyses covering parameters including urea, glucose, uric acid, and cholesterol are feasible [23]. As the requirement for cost-effective and compact analytical tools has risen, biological sensors have become extensively employed for the specific detection of analytes [21,24,25].

The principal challenge in enhancing both selectivity and sensitivity within biosensor system manufacturing lies in maintaining the functionality of immobilized biomolecules. This aspect is significantly influenced by factors such as pH levels, temperature variations, humidity levels, and exposure to substances that may be toxic [22,26,27,28]. In the realm of clinical diagnostics, the paramount parameters for biosensors are sensitivity and selectivity, given their crucial role in furnishing accurate readings. Nanostructures emerge as particularly promising sensing materials in terms of sensitivity for several reasons: (1) the augmented surface area facilitates equivalence in size between nanoparticles and analytes, thereby enhancing sensitivity for diminutive analytes; (2) heightened direct electron transfer contributes to increased sensitivity and a refined limit of detection; and (3) the nanostructure’s particle size, akin to the Debye length, efficiently amplifies sensor sensitivity [29].

An additional critical aspect influencing the effectiveness of biosensors encompasses the nano–bio interfaces established between NMOs and immobilized biomolecules. Furthermore, the judicious selection of the most suitable nanostructured metal oxides tailored for the specific requirements of biomolecule immobilization is integral to optimizing biosensor performance. Several determinants impact the nano-bio interface, encompassing characteristics such as the surface roughness and porosity of NMOs, surface area, charge, valence/conductivity states, functional groups, hygroscopic properties, physical attributes, and other relevant factors [26]. NMOs such as copper, iron, tin, zinc, nickel, cerium, zirconium, titanium, and magnesium demonstrate compelling catalytic properties and superior nanomorphological, biocompatible, functional, and non-toxic properties. The huge quantity of charge on the surfaces of nanostructured metal oxides is responsible for their exceptional electron characteristics. Consequently, all these NMOs have been employed as nanozymes, especially due to their catalytic properties, and have been reported to have potential in the realm of biosensor applications.

### 1.2. Nanostructured Metal Oxides Used as Nanozymes

Nanozymes are artificial enzymes crafted from nanomaterials. Nanozyme systems, serving as direct surrogates for conventional enzymes, effectively replicate catalytic regions of authentic enzymes or incorporate multivalent components for catalytic reactions. These synthetic enzymes, meticulously designed for precise catalytic functions at the nanoscale, are composed of nanomaterials. They emulate the catalytic properties of natural enzymes, demonstrating versatile applications across various scientific and technological domains [14]. The effectiveness of nanozymes is due to the physicochemical properties and features of nanomaterials [30]. For example, the remarkable attributes of nanozymes, such as their capacity for de novo synthesis, adjustable catalytic activity, and durability against environmental factors, establish them as formidable candidates and prospective alternatives to natural enzymes. In the past few years, diverse nanozymes have been identified or engineered, and they are currently applied in tasks related to molecular detection studies, medical treatment areas, and environmental management. The catalytic mechanisms of nanozymes depend on their size, surface modification, surface lattice, and the composition of their parameters [15]. Studies have shown that nanoparticles can mimic many enzymes, such as catalase, oxidase, hydrolase, uricase, peroxidase, halo peroxidase, glutathione peroxidase, methane monooxygenase, and superoxide dismutase. The pivotal consideration lies in the fusion of unique physicochemical properties and catalytic activities resembling enzymes [31,32]. For example, utilizing nanozymes as substitutes for natural enzymes involves preserving or enhancing the inherent characteristics of enzyme-based biosensors.

Accordingly, affinity biosensors incorporating nanozymes present enhanced cost-effectiveness and heightened stability. Therefore, electrochemical affinity biosensors utilizing nanozymes, predominantly characterized by peroxidase-like efficiency and widely employed as catalytic labels, have witnessed a notable proliferation in recent years [32]. On the other hand, contemporary biosensors utilize diverse transducers to convert biochemical occurrences that result from the interactions between a bioreceptor molecule and an analyte into measurable signals. Electrochemical biosensors may be a good choice since they are designed for easy applicability, target analyte selectivity, offer ongoing monitoring and rapid findings, and have the potential for cost-effectiveness and portability. Additionally, the primary categories of electrochemical biosensors include amperometric, conductometric, and potentiometric types [33,34,35].

### 1.3. Electrochemical Biosensors Based on Nanostructured Metal Oxides

At the core of a biosensor lies the foundational notion of the chemical interplay between a fixed biomolecule and a designated analyte, culminating in either the generation or utilization of ions or electrons. This process induces modifications in the quantifiable electrical characteristics of the solution, encompassing alterations in electric current, potential, conductance, and ionic strength. Many hurdles must be solved during the development of biosensors for commercial usage, such as unwilling interference, biological component instability, poor repeatability, or inaccurate results [33,36].

Electrochemical biosensors are analytical tools that integrate the selective properties of biological recognition elements with the heightened sensitivity of electrochemical detection methodologies. This amalgamation enables the detection and quantification of target analytes within biological samples. These types of biosensors have gained significant attention in clinical diagnostic analyses, environmental surveillance, ensuring the safety of food, and other fields due to their numerous advantages over conventional analytical techniques. Electrochemical biosensors are also highly favored in medical diagnosis due to being a rapid and reliable analytical method. These sensors can detect a wide range of biomolecules in blood, tears, saliva, and sweat for purposes such as cancer diagnosis, genetic disease detection, virus, bacteria, IgG detection, glucose detection, etc. [37,38]. They use a triple-electrode system to efficiently transform chemical alterations into electrical signals [22,23]. One of the electrodes in use, which is a working electrode, undergoes modification through the introduction of a molecule designed for specific biological recognition. Upon the binding of an analyte to a biological recognition element, oxidative and reductive reactions are initiated, eliciting alterations in the electrical properties of the system, thereby yielding the sensor signal [39]. The changes in resistance and capacitance are also measured with electrochemical biosensors. This approach yields consistent outcomes in the identification of target molecules.

Electrochemical biosensors offer notable sensitivity and selectivity for target analytes, owing to the precise interactions between the biological recognition element (such as enzymes, antibodies, or DNA) and the analyte of interest. This specificity serves to mitigate potential interference from other compounds within intricate sample matrices, thereby ensuring the attainment of precise and trustworthy measurements. Electrochemical biosensors typically provide rapid response times, allowing for real-time or near-real-time analysis of target analytes. This rapid detection capability is advantageous for applications requiring timely monitoring of dynamic biological processes, such as glucose monitoring in diabetes management or pathogen assessment in infectious disease diagnostics [40,41,42,43]. Moreover, electrochemical biosensors are often cost-effective compared to traditional analytical methods, rendering their extensive deployment attainable within settings constrained by resources, including developing nations. The simplicity of design and fabrication, coupled with advances in microfabrication technologies, has led to the fabrication of low-cost biosensor platforms that offer affordable solutions for various diagnostic and monitoring applications [44,45]. Electrochemical biosensors can be designed to detect multiple analytes simultaneously, allowing for multiplexed analysis of complex sample matrices. Multiplexing capabilities enable the comprehensive profiling of biomarkers or analytes of interest in a single measurement, enhancing diagnostic efficiency and throughput while conserving time and resources. Electrochemical biosensors often exhibit long-term stability and reusability, allowing for repeated measurements over extended periods without important degradation in performance. Stable biosensor platforms enable the continuous monitoring of target analytes in continuous monitoring applications, such as environmental monitoring or long-term disease management [46,47,48,49].

**Figure 2:**
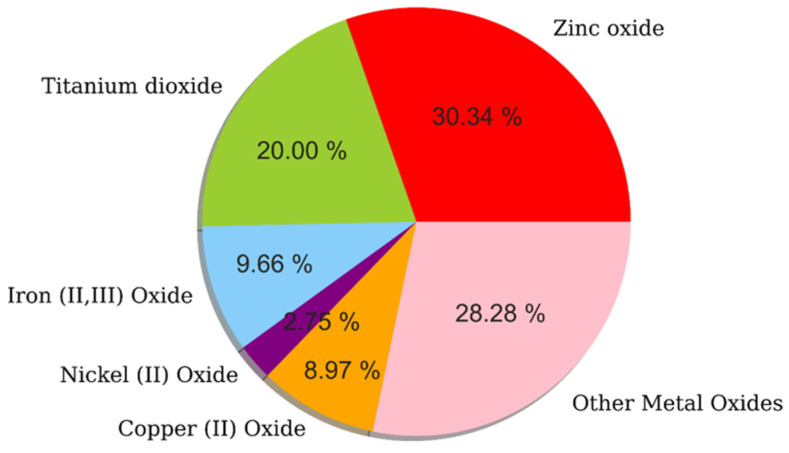
Distribution of nanostructured metal oxides utilized in biosensors for medical diagnosis in the last decade. Data from Web of Knowledge for 2014–2024.

The integration of electrochemical biosensors with labs-on-chips (LOCs) presents an opportunity to develop optimal point-of-care (POC) analytical platforms, given their pragmatic utility, heightened sensitivity, and capacity to deliver prompt results [50]. Additional benefits include the affordability and portability of these types of devices. Furthermore, the incorporation of nanoparticles during fabrication allows a lower detection limit to be achieved. Generally, electrochemical biosensors offer numerous advantages, encompassing heightened sensitivity, swift response rates, miniaturization, portability, affordability, operational simplicity, multiplexing capabilities, and enduring stability. These attributes collectively render them indispensable instruments for an extensive array of diagnostic, monitoring, and analytical undertakings across healthcare, environmental surveillance, food safety, and related domains. Anticipated progressions in biosensor technology and manufacturing methodologies are poised to augment their efficacy, adaptability, and utility across diverse domains in the forthcoming years [38,51,52]. One of the best options that can be used in the improvement of electrochemical biosensors is metal oxide nanoparticles [38]. These include a high surface-to-volume ratio, adjusted surface working function, augmented surface reaction activity, potent catalytic effectiveness, and commendable adsorption capacity [3,4]. Also, the structure, size, and shape of the NMOs influence all of the listed properties (such as mechanic, electric, magnetic, optic, and catalytic properties) of the NMOs. Therefore, their effectiveness can be designed using NMOs such as copper, iron, manganese, zinc, titanium, nickel, cobalt, zirconium, tungsten, silver, and vanadium metal oxide nanoparticles. The selection of these nanoparticles as an immobilizing matrix depends on their morphological structure, biocompatibility, non-toxicity, catalytic properties, orientation, and conformation [53]. Although a wide range of NMOs are available, zinc oxide (ZnO), titanium dioxide (TiO_2_), iron oxide (Fe_3_O_4_), nickel oxide (NiO), and copper oxide (CuO) nanoparticles are reported to be the most preferred ones, seen as attractive in various biosensor technologies due to being relatively safe for mammals to use. Due to their unique features, they attract great attention from researchers in technological fields such as medicine, biomedical, material chemistry, agriculture, information, optics, electronics, catalysis, environment, energy, and sensors [5,6,54,55]. Accordingly, this review discusses these NMOs, their integration into electrochemical biosensors in the medical field, and their analytical performance. Figure 2 shows the percentages of nanostructured metal oxides, and as can be seen from the figure, the most employed nanostructured metal oxide nanoparticles in medical diagnosis in biosensors were ZnO, TiO_2_, and Fe_2_O_3_.

## 2. Applications of Nanostructured Metal Oxides Used in Electrochemical Biosensors for Medical Diagnosis

### 2.1. Zinc Oxide-Based Electrochemical Biosensors

Among NMOs, ZnO nanostructures exhibit distinct advantages. A greater number of analytes can be accommodated due to the elevated surface-to-volume ratio. In this way, higher-sensitivity biosensors can be produced. Moreover, because of their high electron transfer rate, ZnO nanostructures can elicit biomolecules’ hidden electrochemical capacity and promote direct electrochemistry in analytes, especially when their redox capability remains obscured due to the insulation of their redox centers [56]. Noteworthy characteristics of ZnO nanostructures include their elevated catalytic efficiency, high isoelectric point, robust adsorption capability, and broad biocompatibility due to a wide bandgap. Additionally, the diverse properties of ZnO nanostructures contribute to their extensive applications in pharmaceutical analysis, spanning medical diagnosis, food safety investigations, and environmental pollution monitoring [57,58,59,60,61]. In a recent investigation, an Interdigitated Electrode (IDE) was developed on a glass substrate featuring 37 combs. Each comb exhibited a width of 70 μm with a spacing of 100 μm. A slender layer of solution was applied to the device and subjected to spin coating, preheating, and annealing to establish nuclei. Subsequently, the tool was immersed in an aqueous solution comprising Zn (NO_3_)_2_, hexamethylenetetramine, and deionized water, facilitating the synthesis of ZnO nanowires (NWs). The reaction formulas for ZnO NW synthesis in this solution are as follows [18]:

$$C_{6}H_{12}N_{4(S)}+6H_{2}O_{\left(L\right)}↔4NH_{3}+6HCHO$$

$${NH}_{3}+H_{2}O↔NH_{4}^{+}+OH^{-}$$

$${Zn}^{2+}+4NH_{3}↔[Zn(NH_{3}{)}_{4}{]}^{2+}$$

$$Zn^{2+}+2OH↔Zn(OH{)}_{2}$$

$$Zn(OH{)}_{2}↔ZnO_{\left(S\right)}+H_{2}O_{\left(L\right)}$$

Furthermore, ZnO-based electrochemical biosensors provide a versatile platform for pharmaceutical detection. The investigation of pharmaceuticals at low concentrations over a short time period is a top priority for researchers in the pharmaceutical field. Various kinds of ZnO NMs have been suggested for use in analyte detection in the literature. In a study, a ZnO nanostructure-based electrochemical immunosensor featuring immobilized ZIKV-NS1 antibodies on a printed circuit board (PCB) was created to detect the Zika virus (ZIKV), which is indeed an important analyte, particularly due to its potential for causing significant public health concerns. Diagnostic tests for the Zika virus help healthcare providers identify infected individuals, implement appropriate measures to prevent transmission, and provide appropriate care and counseling. The authors of a previous study allowed for a quick POC assessment of ZIKV infection through urine. The biosensor exhibited a broad linear detection range from 0.1 to 100 ng·mL^−1^ [62].

The utilization of plant extracts in the eco-friendly production of nanoparticles constitutes a pivotal facet within the realm of nanotechnology. Another noteworthy study involved the improvement of an economical glucose biosensor, as glucose detection plays a pivotal role in medical diagnostics by facilitating the diagnosis, management, and monitoring of diabetes, detecting hypoglycemia, ensuring glycemic control in critical care settings, optimizing pregnancy outcomes, and supporting research efforts in diabetes prevention and treatment, with the use of green-synthesized ZnO nanoparticles derived from *Zingiber officinale* root suggested. The fixation of glucose oxidase (GOx) on a carbon paste electrode (CPE) designed with zinc oxide (ZnO) was accomplished via cross-linking facilitated by glutaraldehyde. This biosensor exhibits notable characteristics, including a minimal detection threshold of 14.7 μM, a swift response duration of under 1 s, elevated sensitivity at 15.98 µA·mM^−1^·cm^−2^, and strong biological interaction indicated by a Michaelis-Menten constant of 0.99 mM. Additionally, the developed biosensor demonstrated good selectivity towards interfering substances such as ascorbic and uric acid [63].

**Figure 3:**
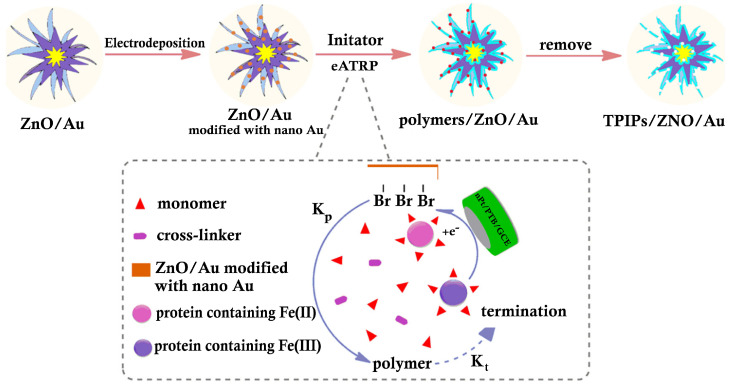
Schematic representation of TPIPs/ZnO/Au preparation. “Reprinted with permission [66] 2019, Elsevier”.

In another study, Sun Y. et al. used electrochemically mediated atom transfer radical polymerization (eATRP) using bovine hemoglobin (Hb), which serves as both a catalyst and template, to construct thermally sensitive protein-imprinted proteins (TPIPs) on the surface of ZnO nanoflowers (Figure 3). Bovine hemoglobin detection is crucial for screening blood products to ensure they are free from contamination with bovine components. Contamination of human blood products with bovine hemoglobin can occur during processing or storage, and it poses a risk of adverse reactions, such as allergic responses or immune-mediated complications, in recipients. Moreover, some individuals may have allergies or sensitivities to bovine proteins, including hemoglobin. Detecting the presence of bovine hemoglobin in diagnostic tests or medical products is important for identifying potential allergens and avoiding adverse reactions in susceptible individuals [64,65]. The range within which the concentration of Hb was linearly measurable was determined to be between 10^−13^ and 10^−1^ mg·L^−1^, with the LOD established at 3.1 × 10^−14^ mg·L^−1^ through the application of differential pulse voltammetry (DPV) [66].

**Figure 4:**
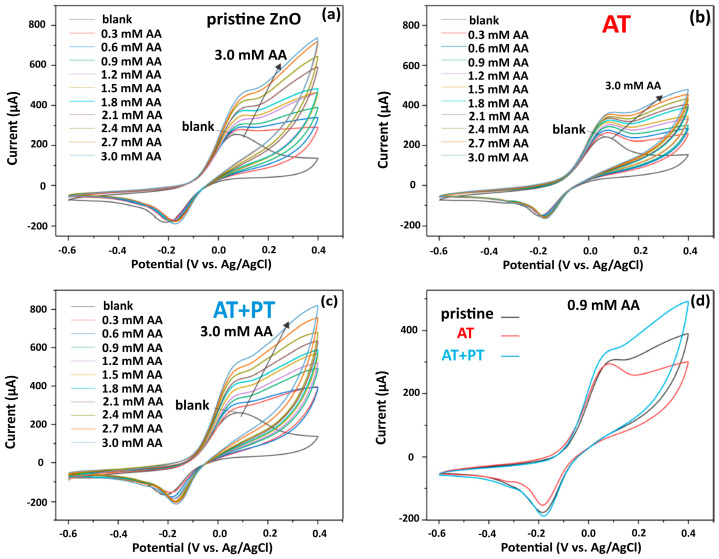
CV responses using a ZnO NW/ITO sensor. (**a**) pristine ZnO sample; (**b**) air-annealed (AT) sample; (**c**) air-annealed followed by hydrogen plasma treatment (AT+PT) sample; (**d**) CV curves of pristine ZnO, AT, and AT+PT samples at AA concentration of 0.9 mM. “Reprinted with permission [71], 2023, MDPI”.

In 2023, Beattoa et al. designed a biosensor for dopamine, which is a neurotransmitter that plays a critical role in various neurological functions, including movement, cognition, mood regulation, and reward mechanisms, using ZnO@Au core–shell nanostructures as a support material for tyrosinase immobilization on screen-printed carbon electrodes. This biosensor allowed the determination of dopamine with a linear range from 0.1 to 500 μM and a detection limit of 86 nM using differential pulse voltammetry [67]. In a recent investigation, a newly developed electrochemical sensor devoid of enzymes, relying on ZnO nanowire arrays synthesized through low-temperature chemical deposition on the ITO surface, was introduced for the determination of ascorbic acid. Ascorbic acid acts as a potent antioxidant, scavenging free radicals and protecting cells from oxidative damage. Monitoring ascorbic acid levels in biological samples, such as plasma or urine, can provide insights into an individual’s antioxidant status and risk of oxidative stress-related diseases, including cancer, cardiovascular disease, and neurodegenerative disorders [68,69,70]. The ascorbic acid sensitivity value was found to be 92 µA·mM^−1^·cm^−2^ for the suggested ZnO-modified sensor (Figure 4) [71].

### 2.2. Titanium Dioxide-Based Electrochemical Biosensors

Pre-precipitated TiO_2_ nanoparticles underwent interaction with a concentrated NaOH solution, leading to the formation of sheet-like sodium titanate. This transformation occurred through either the dissolution or delamination of titania, as shown in the following reactions. Following this, exfoliation from the stacked sodium titanate turned these nanosheets into nanotube-like structures [72,73]. Under hydrothermal conditions, nanosheets, featuring uneven surface energy on their upper and lower sides, undergo scrolling and folding processes to form tubes, whether single- or multilayered. The final products, titanium dioxide nanotubes, are obtained by washing the sodium titanate nanotubes with a dilute hydrochloric acid (HCl) solution (0.1 M) [74,75].

$$[TiO_{2}{]}_{nanoparticle}+2NaOH\to 2{Na}^{+}+TiO_{3}^{2-}+H_{2}O$$

$$2{Na}^{+}+TiO_{3}^{2-}\to [{Na}_{2}TiO_{3}{]}_{nanosheets}$$

$$[{Na}_{2}O_{3}{]}_{nanotubes}\to [{Na}_{2}TiO_{3}{]}_{nanotubes}$$

$$[{Na}_{2}TiO_{3}{]}_{nanotubes}+2H{Cl}_{\left(dil\right)}\to [TiO_{2}{]}_{nanotubes}+2NaCl+H_{2}O$$

Multi-walled carbon nanotubes (MWCNTs), reduced graphene oxide (RGO) and graphene, gold nanoparticles, quantum dots, and various other materials were combined with titanium dioxide nanoparticles to detect substances such as glucose, hydrogen peroxide, bisphenol A, human immunoglobulin G, catechol, chloramphenicol, cyanide, ascorbic acid, and other important compounds in medical diagnostics.

Photocatalytically active TiO_2_ nanoparticles have stable chemical characteristics, minimal toxicity, and a low cost. For photocatalysis, humidity centers, and gas sensing, the utilization of TiO_2_ nanoparticles incorporating metal dopants like gold, platinum, strontium, and zinc has been observed. These doped TiO_2_ nanoparticles have found applications as biosensors, attributed to their excellent chemical stability and enhanced photocatalytic activity. The incorporation of dopants has the potential to enhance both transparency and electrical conductivity, leading to significant modifications in the sensing capabilities and crystalline structure of TiO_2_ nanofilms. Therefore, incorporating doped TiO_2_ nanoparticles has been embraced to improve optoelectronic functionality [72,76,77,78,79]. A heightened sensitive non-enzymatic sensor was fabricated using an electrochemically stable mixed oxide. This includes mesoporous TiO_2_ nanoparticles with defects, along with the surface distribution of Ni^2+^ and Ni^3+^ ions. The introduction of flaws to TiO_2_ nanoparticles using NiO was successful when examining the interfacial characteristics of NiO and TiO_2_. The inclusion of nickel ions within the structure of TiO_2_ nanoparticles promotes effective charge transfer, thereby averting agglomeration during extended detection periods and ensuring notable long-term stability and sensitivity. As a result, this defect-induced mesoporous metal oxide nanocomposite emerged as a promising candidate for utilization as a redox-active material in electrochemical biosensors [80].

**Figure 5:**
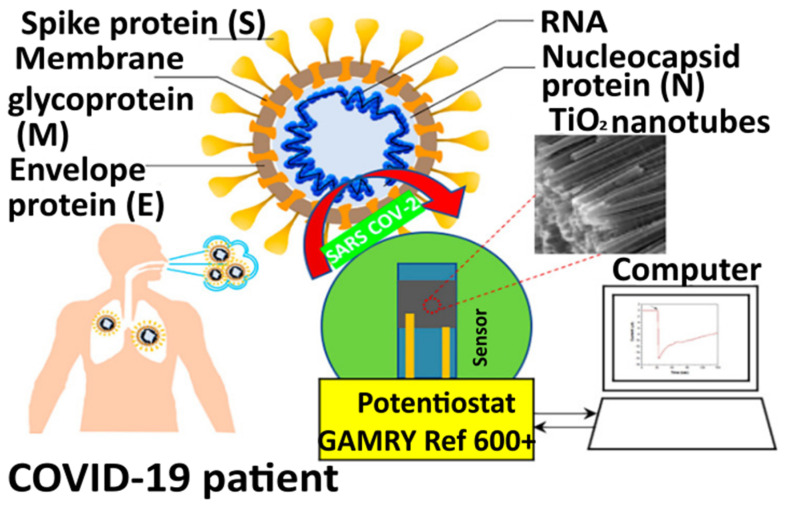
Schematic representation of Co-TNT-based sensor for SARS-CoV-2 detection. “Reprinted with permission [81], 2020, MDPI”.

An economical and sensitive electrochemical sensor was created using cobalt-functionalized TiO_2_ nanotubes (Co-TNTs) to quickly detect SARS-CoV-2. This sensor detects the spike protein, specifically the receptor-binding domain (RBD), on the virus surface. TNTs were produced through a straightforward and inexpensive one-step electrochemical anodization process. Subsequently, the TNT platform underwent cobalt functionalization using an incipient wetting method, and the entire system was connected to a potentiostat for data collection. Notably, this sensor demonstrated the ability to detect the S-RBD protein of SARS-CoV-2 even at deficient concentrations ranging from 14 to 1400 nM. The sensor exhibited a linear correlation in determining viral protein across a broad concentration spectrum. Consequently, the Co-TNT sensor demonstrated notable efficacy in detecting the SARS-CoV-2 S-RBD protein within around 30 s, suggesting its potential application in POC diagnostics for SARS-CoV-2 detection using saliva samples and nasal secretions. Figure 5 illustrates a diagram depicting the direct detection of viruses from a patient sample [81].

In an alternative investigation, solutions of titanium–isopropoxide (TTIP) in ethanol were blended with CeO_2_ nanoparticles to create a mixed sol–gel. Subsequently, upon depositing the resultant TiO_2_-CeO_2_ sol over a glass substrate, the resulting nanocomposite thin film underwent examination for phase composition and for the observation of surface morphology using SEM, TEM, XRD, and Fourier Transform Infrared (FT-IR) methods. The CeO_2_-TiO_2_ sol was efficiently manufactured within a short timeframe using the sol–gel spin coating technique. A biosensor for uric acid was then developed by immobilizing a combination of enzymes (urease and glutamate dehydrogenase) onto the nanocomposite film. This biosensor demonstrated a rapid response time of 10 s, a sensitivity of 0.9016 μA·cm^−2^·mM^−1^, and a LOD of 0.165 mM. Assessment via CV revealed the enhanced sensitivity and expanded linear range of the nanocomposite film. These findings underscore the innovative properties of the combined enzyme and TiO_2_-CeO_2_ material for uric acid assessment in real blood, particularly in the context of arthritic conditions [82].

In a study by Khaliq et al., TiO_2_ nanotubes (TNTs) were made by anodizing titanium (Ti) foils and then using the chemical bath deposition process to adorn them with Cu_2_O NPs. The manufactured electrode had a high catalytic activity for cholesterol oxidation, as measured by CV and amperometric response. When compared to the electrodes, the hybrid electrode showed a 5-fold increase in sensitivity of 6034.04 μA·mM^−1^·cm^−2^. The LOD and rapid response time were found to be 0.05 μM and 3 s, respectively. This research suggests that Cu_2_O NP-decorated TNTs might be used to produce very stable, repeatable, and selective biosensors [83].

In a study conducted by Yadav et al. in 2023, a TiO_2_–guanine nanocomposite (TG NC)-based disposable biosensor was fabricated for the rapid determination of the H1N1 swine flu virus. The rapid and accurate detection of the H1N1 swine flu virus allows for early diagnosis of influenza infection. The developed biosensor displayed high sensitivity, found to be 40.32 μA·ng^−1^·cm^2^, a low LOD of 0.00024 ng·6 µL^−1^, and a wide linear range according to CV and electrochemical impedance spectroscopic (EIS) analysis results [84]. It is very important to diagnose the virus when its concentration is very limited (early detection) before it becomes more severe over time. The LOD value of a TG-NC-based biosensor was found to be very low with respect to the standardized methods in the literature, such as two-step reverse-transcription PCR (50 ng·μL^−1^) and nested PCR (0.001 ng·μL^−1^) [85]. This reveals that the use of NMOs enhances analytical performance, providing early detection of viruses even at negligible concentrations.

### 2.3. Iron (II, III) Oxide-Based Electrochemical Biosensors

The co-precipitation technique involves the combination of ferric and ferrous ions in a 1:2 molar ratio within highly alkaline solutions, either at ambient or elevated temperatures, representing the most prevalent procedural technique. Typically, the reaction is shielded by a gas. When the solution’s pH falls below 11, the formation of the Fe_3_O_4_ nucleus is facilitated, whereas the expansion of the Fe_3_O_4_ nucleus is facilitated when the solution’s pH exceeds 11 [86,87,88,89]. The chemical process is given below:

$${Fe}^{2+}+{Fe}^{3+}+8OHFe(OH{)}_{3}\to Fe(OH{)}_{2}+2Fe(OH{)}_{3}\to {Fe}_{3}O_{4}+4H_{2}O$$

**Figure 6:**
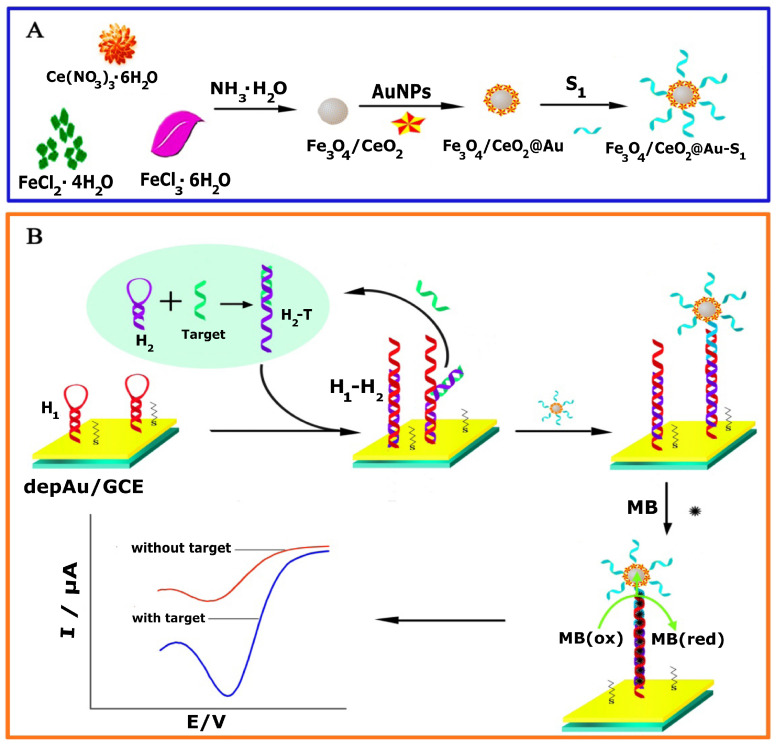
Schematic representation of (**A**) the Fe_3_O_4_/CeO_2_ @Au-S_1_ preparation and (**B**) the detection principle for microRNA-21. “Reprinted with permission from [93], 2018, Elsevier”.

miR-21 is dysregulated in various diseases, including cancer, cardiovascular diseases, neurodegenerative disorders, and autoimmune diseases. Detection of miR-21 limits in biological fluids, such as plasma, blood, or tissues, can serve as a biomarker for disease diagnosis, prognosis, and prediction of treatment response [90,91,92]. In a previous study, the authors proposed an enzyme-free biosensor modified for the detection of microRNA-21, utilizing Fe_3_O_4_/CeO_2_@Au magnetite NPs (Fe_3_O_4_/CeO_2_@Au MNPs) as a nanocatalyst. The biosensor employs a catalytic hairpin assembly for signal processing. To initiate the process, the target microRNA-21 forms a bond with hairpin H_2_, resulting in the formation of H_2_-T double-stranded DNA (dsDNA). Subsequent to this interaction, there exists the potential for the initiation of the unfolding of hairpin H_1_, thereby facilitating the formation of double-stranded DNA (dsDNA) between H_1_ and H_2_. Concurrently, the Fe_3_O_4_/CeO_2_@Au-S_1_ hybridizes not only with single-stranded fragments of H_1_–H_2_ dsDNA, leading to the generation of elongated dsDNA capable of adsorbing a considerable quantity of methylene blue (MB) electroactive species, but also functions as a nanocatalyst, catalyzing the reduction of MB directly. This catalytic process serves to amplify the electrochemical signal, as shown in Figure 6 [93]. Since cerium oxide (CeO_2_) and Au NPs can considerably boost the activity of catalysis for Fe_3_O_4_ NPs and successfully avoid agglomeration of Fe_3_O_4_ NPs, Fe_3_O_4_/CeO_2_@Au MNPs showed good catalytic performance. The suggested biosensor had a wide linear range from 1 fM to 1 nM, a low LOD of 0.33 fM, and outstanding specificity and sensitivity for microRNA-21 detection, thanks to the signal amplification method. This method opened up new possibilities for detecting additional biomarkers in electrochemical biosensors [93].

The determination of prostate-specific antigen (PSA) detection in medical diagnostics is essential for prostate cancer screening, diagnosis, monitoring, risk assessment, and prognostication [94,95]. The prostate-specific antigen biomarker was detected by employing an ultrasensitive electrochemical immunosensor. The biosensor was fabricated through the surface modification of a GCE with a nanocomposite consisting of MWCNTs and Fe_3_O_4_ NPs. MWCNTs with COOH groups were an excellent substrate for modifying the electrode to produce a sandwich-like shape for the binding of the anti-total PSA antibody (Ab_1_). An anti-free PSA antibody (Ab_2_) tagged with HRP resulted in an increase in current with the addition of a larger PSA concentration. The PSA measured by the immunosensor with a linear concentration range of 2.5 pg·mL^−1^–100 ng·mL^−1^ had a LOD of 0.39 pg·mL^−1^ [96].

HBsAg detection in medical diagnostics plays a critical role in the medical diagnosis, screening, prevention, and management of hepatitis B infection, contributing to improved patient outcomes and public health efforts to control HBV transmission and reduce the burden of hepatitis B-related liver disease [97,98]. In a recent investigation, a highly sensitive immunosensor was devised for the identification of the human hepatitis B surface antigen (HBsAg). This immunosensor employed a graphene oxide (GO)/Fe_3_O_4_/Prussian blue (PB) nanocomposite-based electrode. Prussian blue was harnessed as a redox probe within an electrochemical immunoassay setup. Additionally, the fabricated nanocomposites and gold nanoparticles (AuNPs) were synthesized and integrated into screen-printed electrodes to augment detection sensitivity and streamline the immobilization process of the hepatitis B surface antibody (HBsAb). The immunosensor demonstrated keen responsiveness to HBsAg across a concentration band from 0.5 pg·mL^−1^ to 200 ng·mL^−1^, boasting a notably low detection threshold of 0.166 pg·mL^−1^. Manifesting a broad linear range, a minimized detection limit, exceptional biocompatibility, remarkable selectivity, and enduring operational stability, the proposed immunosensor exhibited commendable efficacy in HBsAg identification [99].

In a recent study, screen-printed carbon electrodes (SPCEs) were modified with Fe_3_O_4_-Au core–shell NPs. Then, the label-free biosensor was fabricated with a thiolated single-strand DNA (ssDNA) probe belonging to human papillomavirus (HPV) DNA sequences. The linear range, the LOD, and the sensitivity of the HPV biosensor were found by DPV to be 10^−4^–1 μM, 0.1 nM, and 2.4 μA·nM^−1^, respectively [100]. In a study conducted by Ren et al. (2023), iron oxide magnetic nanoparticles with aptamers (sDNA-Fe_3_O_4_ MNPs) were used as immobilizing agents onto the surface of the UIO-66-NH_2_ carrier container for quantitative detection of amyloid-beta oligomers (AβO), which serves as a disease marker in Alzheimer’s disease. The sensor displayed improved signals in differential pulse voltammetry across an expansive linear range from 10 fM to 10 μM, with a low LOD of 3.4 Fm [101].

**Figure 7:**
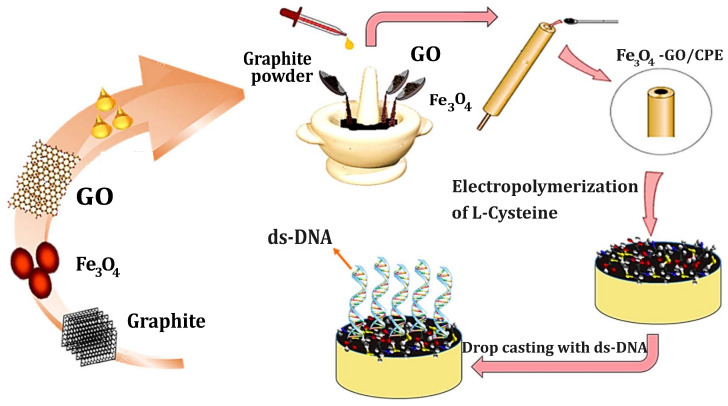
Design of the ds-DNA/p(L-Cys)/Fe_3_O_4_ NPs-GO/CPE biosensor. Reprinted with permission from [102], 2018, Elsevier.

In another study, the goal was to develop a basic and efficient DNA biosensor based on a CPE designed with ds-DNA/poly(L-cysteine)/Fe_3_O_4_ NPs and GO (ds-DNA/p(L-Cys)/Fe_3_O_4_ NPs-GO/CPE) for the sensitive determination of guanine and adenine, whose detection can help diagnose genetic disorders and inherited diseases, such as cystic fibrosis, sickle cell anemia, Huntington’s disease, and various forms of cancer. Moreover, the detection of adenine and guanine mutations in oncogenes and tumor suppressor genes is crucial for cancer diagnosis, prognosis, and treatment. Genetic testing for specific mutations, such as the BRAF V600E mutation in melanoma or EGFR mutations in lung cancer, helps identify targeted therapies that selectively inhibit cancer cell growth and improve patient outcomes. Analyzing adenine and guanine alterations in cancer genomes also provides insights into tumor heterogeneity, evolution, and drug resistance mechanisms, guiding the development of new cancer therapies. In this study, DPV and CV were employed to observe the electrocatalytic oxidation of guanine and adenine on the electrode. Peak currents and electron transfer kinetics for the oxidation reactions of adenine and guanine increased in the ds-DNA/p(L-Cys)/Fe_3_O_4_ NPs-GO/CPE. In contrast, the over potential for the oxidation reactions of the targets decreased. The linear concentration ranges for the targets were 0.01–30.0 μM and 0.01–25.0 μM were found for adenine and guanine, respectively. Moreover, LOD values of 3.90 nM and 1.58 nM were found for adenine and guanine, respectively [102]—a schematic diagram illustrating the development process of the modified electrode is shown in Figure 7.

The Fe_3_O_4_ nanozyme was the first NP with inherent peroxidase-like activity to be discovered, and it has since been extensively employed in biomedicine. Histidine residues were added to a Fe_3_O_4_ NP surface to improve its catalytic action by simulating the enzymatic milieu of natural peroxidase enzymes. The results revealed that a single amino acid change improved the Fe_3_O_4_ nanozyme’s apparent affinity (Km) for the substrate H_2_O_2_ by more than ten-fold and increased its catalytic efficiency (Kcat/Km) by a factor of twenty. By increasing the affinity for peroxidase, Histidine increased the peroxidase-like activity and catalytic effectivity of the Fe_3_O_4_ nanozyme. By enhancing the binding affinity for H_2_O_2_ through the formation of hydrogen bonds between the imidazole group of Histidine and H_2_O_2_, which mimics the configuration of the active site of HRP, Histidine augmented the peroxidase-like activity and catalytic efficiency of the Fe_3_O_4_ nanozyme. Additionally, for peroxidase-like activity, Histidine alteration boosts catalase-like activity, which reflects the increased attraction of H_2_O_2_ during the first reaction step [103,104].

In a very recent study, our research team introduced an innovative technique for the swift on-site identification of tannic acid (TA), a prevalent organic pollutant encountered across diverse natural settings, notably deriving from botanical origins. The proposed method entails the fabrication of a compact electrochemical sensor incorporating a nanozyme framework. This framework encompasses iron oxide nanoparticles (Fe_2_O_3_ NPs) embedded within a chitosan (Ch) substrate, immobilized onto a sulfur-doped graphene (S-Gr) substrate affixed to a gold electrode (AuE). The Fe_2_O_3_ NPs demonstrated peroxidase-like artificial enzyme characteristics, enhancing both stability and catalytic efficacy in the TA oxidation procedure. The amalgamation of these advanced nanomaterials with a microfabricated electrode presents an economically feasible, dependable, and efficient solution for TA detection, with potential applicability in large-scale environmental monitoring initiatives. Furthermore, the Ch matrix serves as a stabilizing agent, augmenting the operational performance and reusability of the nanozyme, while the S-Gr substrate facilitates expeditious electron transfer, culminating in heightened sensitivity and rapid response capabilities. The devised Fe_2_O_3_–Ch-S-Gr/AuE sensing platform demonstrates a low LOD of 3.6 × 10^−3^ µM along with heightened sensitivity, encompassing a broad linear concentration range for TA detection. Selectivity evaluations corroborate the sensor’s precision in discriminating TA amidst potential interfering entities, underscoring its resilience in environmental surveillance contexts. These advancements hold considerable promise in redefining the landscape of environmental monitoring capabilities [105].

### 2.4. Nickel (II) Oxide-Based Electrochemical Biosensors

Nickel oxide (NiO) is a p-type semiconductor, meaning that positive holes carry the current. Nickel oxide is rarely stoichiometric (i.e., Ni1.00O1.00), and the crystal lattice contains a tiny number of Ni^3+^ ions in addition to Ni^2+^ ions. The conductivity of the oxide is directly correlated with the concentration of Ni^3+^ ions within the lattice. The equilibrium is given below:

$${2Ni}^{2+}+{1/2\;O}_{2}↔{2Ni}^{3+}+O^{2-}$$

This indicates that when the partial pressure of oxygen rises, so does the concentration of Ni^3+^ in NiO, resulting in increased conductivity [106,107,108].

To exemplify nickel (II) oxide-based electrochemical platforms for non-enzymatic glucose detection, the development of three-dimensional (3D) NiO structures, such as NiO nanosheets or nanospheres, has been noted for its remarkable electrocatalytic activity. The distinct zigzag arrangement of NiO within graphene oxide and the development of a 3D porous nanostructure represent notable characteristics of these platforms. The redox interactions occurring between Ni^2+^ ions on the surface of NiO and glucose molecules under electrochemical conditions elucidate the mechanism by which NiO nanoparticles come into contact with glucose. In an alkaline solution, Ni^2+^ ions undergo electrochemical oxidation to Ni^3+^. Consequently, NiO has been devised for glucose detection without the necessity of enzyme involvement [109].

In a study by Xiao et al., the direct carbonization of bimetallic Cu/Ni-based MOF (Cu/Ni-MOF) resulted in metal–metal oxide (M-MO) NPS being distributed well throughout the porous carbon matrix, resulting in a composite (M-MO@C). M-MO@C-800 demonstrated outstanding glucose-sensing capability with a larger linear range of 0.1–2.2 mM and a lower LOD of 0.06 mM due to a synergistic benefit from Cu_2_O/CuO, Ni/NiO, and porous carbon. In addition, M-MO@C-800 has high selectivity, outstanding repeatability, and excellent stability. The successful detection of glucose in real samples revealed that M-MO@C might be used as a good candidate glucose sensor in the future [110].

In the context of medical diagnostics, the detection of acetylcholine holds significant importance due to its role as a neurotransmitter essential for the functioning of the central and peripheral nervous systems. Acetylcholine acts as a vital chemical messenger in transmitting nerve impulses across synapses, facilitating communication between neurons and target cells. Monitoring acetylcholine levels enables the assessment of the integrity and efficacy of cholinergic neurotransmission, which is critical for various physiological processes, including cognition, memory, muscle contraction, and regulation of the autonomic nervous system. This study focused on acetylcholine (ACh) detection; a field-effect transistor was engineered using carbon nanotubes (CNTs) and enzymes. The device was meticulously designed, featuring an indium tin oxide-coated glass plate as the substrate, ZnO as the bottom insulator, K-doped CNT as the n-type channel, drain, and source regions, ZrO_2_ as the top gate insulator, and chitosan/nickel oxide (CH/NiO) nanocomposite as the sensing membrane. Acetylcholine esterase (AChE) was immobilized on the sensing membrane through physical adsorption. The experimental results demonstrated excellent linearity and sensitivity, with a sensitivity value of 58 mV/decade observed for ACh concentrations ranging from 0.01 to 0.2 mM [111].

Lactate detection in medical diagnostics provides valuable information about tissue perfusion, cellular metabolism, organ function, and clinical status in a wide range of medical conditions, guiding diagnosis, treatment, and prognostication in critically ill patients and athletes alike [112,113,114,115]. In a recent study performed by Arivazhagan and Maduraiveeran, in 2023, Au@NiO nanodentrite microarrays were developed as microsensors for the electrochemical determination of lactate and glucose. Depending on CA measurements, the microsensor depicted a wide linear range that was found to be from 10.0 μM to 5.0 mM with the LOD value of 100.0 nM for glucose, and a broad linear range of 100.0 μM to 10.0 mM with the LOD value of 8.2 μM for lactate. The sensitivity was calculated to be 11.89 and 11.46 μA·mM^−1^·cm^−2^ for lactate and glucose, respectively [116]. In a recent study, Khorablou et al. developed an electrochemical aptasensor consisting of a combination of nickel oxide nanoparticles (NiONPs) and MXene to detect Methamphetamine (MAMP), a highly addictive recreational drug. Its detection plays a crucial role in identifying substance abuse, assessing acute toxicity, managing psychiatric symptoms, monitoring maternal–fetal health, supporting forensic investigations, contributing to improved patient outcomes, and leading public health efforts to address substance use disorders [117,118]. The sensing platform detected the MAMP with a low LOD of 333.3 fM and a broad linear range from 1 pM to 50 mM [119].

A new oxidase-mimicking nanozyme, NiO, was examined in another study. NiO is unique in that it can oxidize fluorogenic amplex red very well under physiological conditions, making it helpful for intracellular imaging. For nanozyme and immunoassays, amplex red is the most frequently utilized fluorogenic substrate. H_2_O_2_ was necessary for the majority of previously observed amplex red oxidation, which relied on its peroxidase activity. Due to its minimal background interference and good sensitivity in fluorescence detection, this technique is anticipated to be extensively utilized in key bioanalytical applications [120].

### 2.5. Copper (II) Oxide-Based Electrochemical Biosensors

Glucose can be detected with Cu-based electrochemical biosensors. Fourth-generation glucose sensors (FGGS) are the most effective for glucose detection [121]. Copper-based functionalized graphene-gold nanocomposites (FGGS), akin to metal oxide-based non-enzymatic glucose sensors (NEGS), demonstrate efficacy under diverse pH environments. The operational principle of these sensors hinges upon the activation of the metal oxide interface, facilitated by the presence of highly reactive hydroxide ions, which concurrently act as catalysts in the oxidation process of glucose molecules. Utilizing copper oxide-based NEGS, a mechanistic approach for glucose sensing was developed [121,122,123].

$$CuO+OH^{-}\to Cu{\left(OH\right)}_{\left(2\right)}+e^{-}$$

$$Cu{\left(OH\right)}_{2}+OH^{-}\to CuOOH+H_{2}O+e^{-}$$

$$CuOOH+C_{6}H_{12}O_{6(glucose)}\to Cu{\left(OH\right)}_{2}+C_{6}H_{10}O_{6(gluconolactone)}$$

One study used the hydrothermal approach to make hierarchical CuO nanosheets in high quantities. Various methods were used to observe the morphological, structural, and optical attributes of the hierarchical CuO nanosheet sample as it was prepared. Engineered hierarchical CuO nanosheets were used to construct an electrochemically based non-enzymatic glucose biosensor. CV and amperometry (*i–t*) methods investigated the electrochemical performance of the generated biosensor towards glucose. The non-enzymatic biosensor had a high sensitivity of 1467.32 μA·mM^−1^·cm^−2^. The linear range was calculated to be from 0.005 to 5.89 mM, and the rapid response time and LOD were calculated to be ~3.5 s and 12 nM for glucose detection, respectively [124]. Another study used a co-precipitation approach to synthesize the ZnO-CuO nanocomposite (NC) and examined it utilizing XRD, FT-IR, Raman spectroscopy, and Field Emission Scanning Electron Microscopy (FESEM) methods. The anti-LPS *E. coli* antibody was physisorbed after the ZnO–CuO NC was screen-printed on gold-plated electrodes to construct the immunosensor. The sensitivity was determined to be 11.04 μA·CFU^−1^. The LOD was determined to be 2 CFU·mL^−1^ with a linear detection range of 10^3^ to 8 × 10^4^ CFU·mL^−1^ [125].

**Figure 8:**
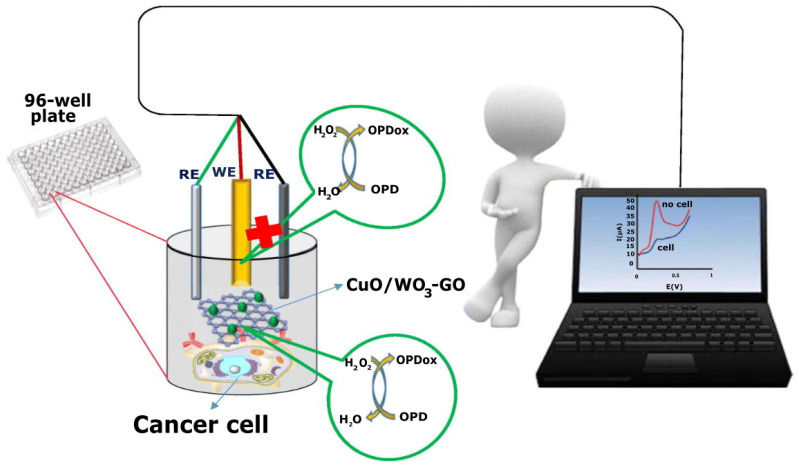
The schematic diagram for the CuO/WO_3_-GO sensor. “Reprinted with permission from [126], 2019, Elsevier”.

The catalytic reaction of H_2_O_2_ based on *o*-Phenylenediamine (*o*PD), a new assessment for the electrochemical detection of cancer cells, was developed employing CuO/WO_3_ modified graphene oxide nanosheet (CuO/WO_3_-GO) with improved peroxidase like-activity (Figure 8). A compact electrochemical apparatus was devised for the determination of cancer cells, employing the synthesized nanocomposite in conjunction with folic acid (FA) as a targeting ligand. In this technique, *o*PD might oxidize on the surface of the working electrode in the presence of H_2_O_2_, resulting in an electrochemical signal.

A chemical reaction involving the H_2_O_2_–*o*PD system occurred and was removed from the electrode, while the interaction between cells and CuO/WO_3_-GO led to a decrease in the signal. The CV approach was used to discover electrochemical characteristics. As a result, cancer cells were discovered at a LOD of 18 cells·mL^−1^ and a detection range of 50 to 10^5^ cells·mL^−1^ [126].

A non-enzymatic metal oxide (CuO-MgO) NC was reported to exhibit effective dopamine detection. A scalable sol-gel technique was utilized for the controlled development of a CuO-MgO nanocomposite. Raman spectroscopy, XRD, and TEM characterization were employed for structural, elemental, and morphological studies. CV and CA methods were used to investigate the electrocatalytic behavior of CuO-MgO in dopamine determination. The sensitivity of the CuO-MgO nanocomposite catalyst was 69 μA·mM^−1^·cm^−2^, and the LOD was calculated to be 6.4 μM in the linear range of 10–100 μM [127].

**Figure 9:**
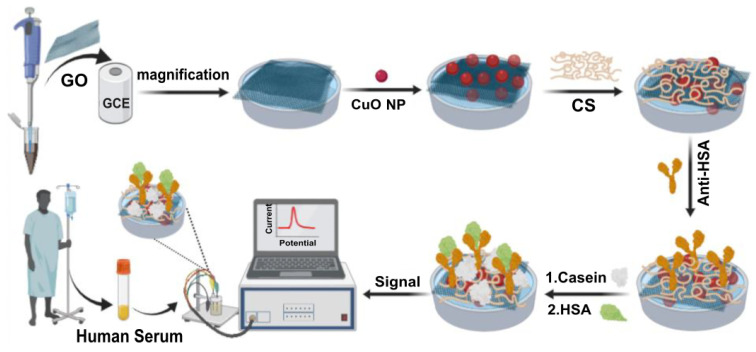
Design of HSA/Casein/Anti-HSA/Glu/CS/CuO NPs/ERGO/GCE human serum albumin immunosensor. “Reprinted with permission from [130], 2020, Elsevier”.

Human serum albumin (HSA) is indeed an important analyte in clinical diagnostics and research. It is the most abundant protein in human blood plasma. It plays crucial roles in maintaining osmotic pressure; transporting various substances such as hormones, fatty acids, and drugs; and acting as an antioxidant. Changes in HSA levels or function can indicate various physiological and pathological conditions, such as liver or kidney diseases, malnutrition, inflammation, or certain cancers. Alterations in HSA concentration or structure are associated with numerous diseases and medical conditions, making it a valuable biomarker for diagnostic purposes. Monitoring HSA levels can aid in disease diagnosis, prognosis, and treatment monitoring. For clinical relevance, the detection limit of a sensor should ideally be within the clinically relevant concentration range of HSA in human blood, which typically falls within the range of tens of milligrams per milliliter. Sensors with detection limits in this range or even lower are considered suitable for clinical applications, as they can accurately measure HSA levels within the physiological range and detect changes associated with disease states. The typical range for HSA concentration in blood serum is 35–50 g·L^−1^. Yet, reduced levels of albumin in serum (hypoalbuminemia, <30 g·L^−1^) during illnesses can indicate many diseases [128,129]. Human serum albumin was detected in both standard solutions and serum samples using a new electrochemical immunosensor. The GCE was decorated in three stages: GO was first drop-cast onto the GCE surface and then electrochemically reduced. Second, two phases of in situ electrochemical deposition of CuO NPs were completed. Finally, anti-HSA antibodies were immobilized utilizing chitosan amino groups that had previously been dropped (Figure 9). Common electrochemical and imaging methods were utilized to analyze the surface shape and composition of the modified electrode. The immunosensor’s response was linear in the range of 10–450 ng·mL^−1^ HSA using DPV, with a LOD of 2.6 ng·mL^−1^ [130].

Creatinine detection in medical diagnostics is essential for assessing kidney function, diagnosing and monitoring kidney diseases, guiding drug dosing and therapeutic management, and optimizing perioperative care, contributing to improved patient outcomes and quality of care in clinical practice [131,132]. Kumar et al., 2023 engineered a novel enzymeless electrochemical creatinine biosensor utilizing zwitterion-functionalized Cu_2_O NPs. The sensor was modified by drop-casting single-crystalline Cu_2_O NPs onto the SPCE. The fabricated biosensor demonstrated a linear detection range of 10–200 μM with a 5.0 μM LOD value for creatinine concentration [133]. In a recent study by Bozdoğan, a pencil graphite electrode (PGE) was electrochemically decorated with CuONPs to detect testosterone, a doping test biomarker for facilitating human physical performance. The LOD and linear range of the offered sensor were calculated at 4.6 nM and 5–200 nM, respectively, using square wave adsorptive stripping voltammetry (SW-AdSV) measurements [134].

In their study, Narasimhappa et al. fabricated a tyrosinase-functionalized Cu_2_O NP electrode for the sensitive determination of dopamine (DA). Cu_2_O NPs were synthesized and characterized through the hydrothermal technique using *Artemisia absinthium* leaf extract. DPV and CV methods were employed to investigate the electroanalytical performance of DA at Tyr/Cu_2_O NPs/GCE-modified electrodes. Furthermore, EIS was utilized to assess the interfacial resistance of electron transfer at the electrode. Moreover, a comprehensive investigation of various interfering substances was conducted individually at concentrations of 12.5 µM, including sucrose, zinc ions, ferric ions, phosphate ions, cupric ions, ammonium ions, cysteine, alanine, citric acid, aspartic acid, Histidine, glucose, and tryptophan, in phosphate-buffered solution using DPV. Real sample analysis was performed using dopamine hydrochloride injection. The developed electrode demonstrated a lower LOD of 0.3 µM and an extensive linear concentration range spanning from 10 to 70 µM [135].

Additionally, in a separate investigation, carbon nanofibers (CNFs) modified with Mn_3_O_4_/NiO NPs, which demonstrated synergistic interactions, were observed to enhance electrocatalytic properties for glucose oxidation. SEM and XRD techniques were used to investigate the morphology of surface and molecular size, as well as the crystal structure of the materials, respectively. High-resolution transmission electron microscopy (HRTEM) was further utilized to analyze the microscopic crystal structure of the samples. FT-IR was utilized to examine the chemical structure, while X-ray photoelectron spectroscopy (XPS) was employed to qualitatively analyze the surface components of the materials. CV was used for electrochemical investigations. The LOD and sensitivity of Mn_3_O_4_/NiO/CNFs/GCE were found to be 0.73 µM and 243.74 µA·mM^−1^·cm^−2^, respectively. Moreover, human blood serum samples with varying concentrations of glucose were analyzed utilizing the standard addition technique, yielding favorable recovery results. The developed sensor boasts an impressive linear range, a low LOD, and excellent resistance to interference, suggesting its potential for clinical diagnostic applications [136].

### 2.6. Other Nanostructured Metal Oxide-Based Electrochemical Biosensors

Besides these metal oxide nanomaterials, in the literature, there exist many other types of metal oxides used in medical diagnostic applications of biosensors. To illustrate, Nguyet et al. developed a novel electrochemical label-free DNA biosensor employing a core–shell cerium oxide nanorod@polypyrrole (CeO_2_-NR@Ppy) nanocomposite for the determination of Salmonella. Salmonella detection in medical diagnostics plays a crucial role in food safety, medical diagnosis, public health surveillance, environmental monitoring, and One Health initiatives, contributing to the prevention and control of Salmonella infections and associated diseases in human and animal populations. The synthesis of the CeO_2_-NR@Ppy nanocomposite involved the in situ chemical oxidative polymerization of pyrrole monomers on CeO_2_-NRs, thus establishing a conducive platform for biosensor construction. The electrochemical responses of the biosensor were evaluated through CV and EIS utilizing a redox probe. Optimal conditions yielded a linear detection range of 0.01–0.4 nM, and LOD and LOQ values were found to be 0.084 and 0.28 nM, respectively. The biosensor demonstrated promising results in determining real Salmonella samples [137].

In 2023, a study presented the improvement of an ultrasensitive electrochemical biosensing platform for the determination of swine flu using Serum Amyloid A (SAA) as a biomarker. Nanostructured zirconia-embedded mesoporous carbon nanosheets (nZrO_2_@PC) were synthesized and functionalized with 3-aminopropyl triethoxysilane (APTES). The resulting material (APTES/nZrO_2_@PC) was deposited in ITO, followed by the immobilization of monoclonal anti-SAA antibodies and bovine serum albumin. Morphological and structural analyses confirmed the successful fabrication of the electrodes. Electrochemical characterization showed a high sensitivity of 95.88 μA [log (μg·mL^−1^)]^−1^ cm^−2^) with a linear detection range of 10–100 μg·mL^−1^. The biosensor exhibited excellent performance in the determination of SAA in spiked serum samples, showing good agreement with standard samples. This biosensor, with a shelf life of 28 days, utilizes the synergistic effect of nZrO_2_ and PC nanosheets, providing a wide surface area for enhanced redox activity. This study suggests the potential of this biosensor for various diagnostic detections and proposes further exploration for POC applications [138].

Furthermore, in a separate investigation, advancements were made in the enhancement of electrochemical sensors designed for the assessment of volatile organic compounds (VOCs), specifically targeting acetone and toluene, recognized as biomarkers for lung cancer. Nanocomposites of tin dioxide (SnO_2_) doped with transition metal ions were synthesized, employing the hydrothermal method to achieve the selective detection of these biomarkers. Diverse characterization methods, including XRD, FESEM, UV–visible spectroscopy, and FT-IR, were used to scrutinize the morphological, structural, and compositional aspects of the synthesized materials. The findings revealed a reduction in bandgap following metal ion doping, thereby enhancing charge transfer capability and electrochemical performance. The selective chemisorption of biomarkers onto the nanocomposites resulted in a heightened response characterized by broad linear detection ranges spanning from 20 to 100 ppb for toluene and 1 to 1000 ppb for acetone. Notably, the nanocomposites demonstrated a remarkable specificity towards acetone and toluene, exhibiting detection limits below permissible thresholds. The investigation suggests that doped SnO_2_ nanocomposites exhibit significant potential for the expeditious and precise diagnosis of lung cancer through the identification of diverse VOCs [139].

Sandil et al. investigated a label-free electrochemical immunosensing platform for detecting the cardiac biomarker troponin I (cTnI), utilizing tungsten trioxide nanorods (WO_3_ NRs). WO_3_ NRs were synthesized through low-temperature hydrothermal methods and functionalized with APTES for the covalent immobilization of cTnI antibodies. Structural and morphological analyses were conducted using various spectroscopic techniques. The immunosensor demonstrated a high sensitivity of 6.81 KΩ mL·cm^2^ within a linear concentration range of 0.01–10 ng·mL^−1^, exhibiting excellent reproducibility, selectivity, and long-term stability. The electrochemical characteristics were evaluated through CV and EIS. Selectivity was tested against interfering biomarkers, and shelf life was assessed over 5 weeks. This study underscores WO_3_ NRs’ potential for developing integrated and portable POC diagnostic tools for cardiac determination [140].

In 2023, another investigation introduced an enzymatic glucose biosensor incorporating a magnesium oxide (MgO) film for potential measurement. Glucose oxidase (GOD) was immobilized on the working electrode using APTES GA, with a Nafion layer enhancing glucose selectivity. The sensor utilized a flexible printed circuit board (FPCB) substrate with MgO deposited via radio frequency (RF) sputtering. Morphological analyses were conducted through FESEM, while X-ray photoelectron spectroscopy was employed to detect the composition of MgO. The sensor employed potentiometric measurement via the time–voltage method, featuring a linear glucose detection range of 2 to 10 mM. The experimental findings exhibited superior performance in terms of sensitivity, linearity, response time, interference, and limit of detection compared to conventional biosensors. Glucose selectivity was confirmed through analysis with and without added enzymes. This investigation underscores the potential applicability of the proposed biosensor for glucose detection in medical monitoring and diagnosis [141]. Some selected studies about nanostructured metal oxide-based electrochemical biosensors in medical diagnosis are tabulated in Table 1 with information about the NMOs used, their morphology, the technique for the electrochemical biosensing, electrode type, and other analytical characterizations (Table 1).

## 3. Discussion and Future Directions

NMOs are synthesized in the forms of nanoflowers, nanowires, nanoparticles, nanotubes, nanosheets, hollow spheres, nanoparticles, and nanodentrites. These nanostructures are attached to screen-printed electrodes, indium tin oxide, gold, platinum, and glassy carbon electrodes to develop biosensors. These NMO-based electrochemical biosensors can detect analytes at the fM level as well as in a linear range up to mM levels by cyclic voltammetry, amperometry, differential pulse voltammetry, and electrochemical impedance spectroscopy techniques. NMOs are a highly promising candidate in terms of sensitivity due to their exceptional electron characteristics caused by the massive amount of charge present on their surfaces. Biosensors with greater sensitivity can be designed with ZnO nanostructures due to their elevated surface-to-volume ratio.

Biosensing platforms developed using NMOs for medical diagnosis have attracted the attention of researchers. NMO-based electrochemical biosensors hold immense promise for the future of medical diagnosis. Their remarkable sensitivity, selectivity, and compatibility with nanomaterials present promising opportunities. Recently, NMOs used as nanoparticles have been used to design enzymatic biosensors, genosensors, immunosensors, and cytosensors for the detection of infectious diseases, numerous biomarkers (in genetic, autoimmune, and cancer), small molecules, and so forth. These biosensors exhibit potential in various medical applications, offering the rapid and precise detection of biomarkers associated with diseases. In particular, quantum dots and iron oxide nanoparticles are extensively utilized as diagnostic imaging materials due to their fluorescent and magnetic behavior, respectively. Labeling cells, bacteria, individual molecules, or any biological material with fluorescent NMOs provides reliable quantification of the disease since it allows more obvious visualization. On the other hand, magnetic imaging techniques, generally based on either iron oxide or ferrites, provide timely diagnosis and prognosis of pathological states and diseases such as cancer. Recent advancements have shown that NMOs are often combined with other nanomaterials, such as carbon nanotubes or graphene, to create hybrid structures with enhanced conductivity and stability. This integration improves sensor performance, opening avenues for advanced diagnostic tools. The prospect of point-of-care applications is particularly compelling, as miniaturized and portable biosensors enable on-site diagnostics, which is especially beneficial in resource-limited settings. The continuous refinement of fabrication techniques and materials will likely address challenges related to stability and reproducibility.

Future research may focus on achieving multiplexed detection, enabling simultaneous analysis of multiple biomarkers. The incorporation of these biosensors into wearable and implantable devices has the potential to revolutionize real-time health monitoring, thereby contributing significantly to personalized and preventive medicine. NMO-based electrochemical biosensors represent a transformative trajectory in medical diagnostics, with continual advancements and research poised to sculpt the future landscape of healthcare. Moreover, the development of portable and miniaturized electrochemical biosensors based on NMOs has opened avenues for point-of-care diagnostics. These devices offer rapid and on-site detection, making them valuable tools in resource-limited settings. Ensuring the long-term stability and reproducibility of NMO-based biosensors remains a challenge. Further research is needed to address issues related to sensor degradation over time and batch-to-batch variability. Moreover, the integration of multiple sensing elements in a single biosensor for simultaneous detection of multiple biomarkers is an area of ongoing research. Achieving multiplexed detection with high specificity and sensitivity is crucial for advancing the diagnostic capabilities of these biosensors. As research progresses, these biosensors are poised to play a pivotal role in advancing personalized medicine and improving healthcare outcomes.

Depending on their antimicrobial, antifungal, and antiviral properties, NMOs found other potential application areas, such as tissue and immunotherapeutics, dentistry, regenerative medicine, and wound healing. The biological effects and cytotoxicity of NMOs used for therapeutic purposes should be considered in the context of long-term health risks.

## 4. Conclusions

In recent years, due to developments in the field of nanotechnology, interest in metal oxide nanomaterials has been increasing since they have desirable adsorption properties such as a larger surface area and higher porosity. Their unique properties enable the development of highly sensitive, selective, and rapid detection platforms for a wide range of biomolecules. Many metal oxides are biocompatible, making them suitable for interfacing biological systems. This characteristic is crucial for the development of biosensors that can be employed for in vivo diagnostics and monitoring. Fe_3_O_4_, ZnO, TiO_2_, NiO, and CuO are the most attractive metal oxides due to their unique properties and wide applications.

NMOs are also known as nanozymes due to their ability to mimic enzymes. Nanozymes, which are enzyme mimics, perform well in high-pH and -temperature environments. Many catalytic functions of metal-based nanozymes have been explored and published, including peroxidase, oxidase, catalase, and superoxide dismutase. As discussed and stated in this review, NMOs can serve as excellent platforms for immobilizing enzymes and enhancing the catalytic activity and stability of a biosensor. Enzymatic biosensors based on metal oxides have been employed to detect glucose, cholesterol, and other biomarkers associated with various diseases. On the other hand, NMOs facilitate the immobilization of DNA probes and antibodies, enabling the specific recognition of nucleic acids or proteins. This has applications in the diagnosis of genetic disorders, infectious diseases, and cancer. Many studies are also available in the literature about the use of NOMs in the determination of uric acid, dopamine, and lactate, as well as in the detection of pathogens and hormones. In addition, these nanostructures are frequently used not only in medical analyses but also in food and environmental analyses, water purification, sensor technologies, and increasing energy efficiency.

## Figures and Tables

**Table 1:** Some selected studies about nanostructured metal oxide-based electrochemical biosensors in medical diagnosis.

Morphology of NMO	Technique	Transducer	Analyte	Sensitivity	LOD	Linear Range	Ref.
Zinc Oxide-Based Electrochemical Biosensors							
Nanoflower	DPV	TPIPs/ZnO/Au	Hemoglobin	NS	3.1 × 10^−14^ mg·L^−1^	10^−13^–10^−1^ mg·L^−1^	[66]
Nanoparticle	DPV	ZnO@Au/Tyr/SPCE	Dopamine	NS	86 nM	0.1–500 μM	[67]
Nanowire	CV	ZnO AT+PT/ITO	Ascorbic acid	92 μA·mM^−1^·cm	NS	NS	[71]
Quantum dot	CV	Nf/ZnO QDs/Urs/SPE	Uric acid	4.0 μA·mM^−1^·cm^−2^	22.97 μM	1 mM–10 mM	[142]
Nanorod	AMP	Nf/GOx/ZnO NRs/ITO	Glucose	48.75 μA·mM^−1^·cm^−2^	0.06 mM	0.05–1 mM	[143]
Nanowire	AMP	GOx/ZnO-NWs/Gr	Glucose	13–17 μA·mM^−1^·cm^−2^	3–13 μM	NS	[144]
Nanoporous	EIS	FTO/ZnO/Urs	Urea	0.0506 kΩ·mg ·dL^−1^	5.0 mg·dL^−1^	8.0–110 mg·dL^−1^	[145]
Titanium Dioxide-Based Electrochemical Biosensors							
Nanoparticle	CV	NiO-TiO_2_/GCE	Glucose	24.85 µA·mM^−1^·cm^−2^	0.7 µM	2 µM–2 mM	[80]
Nanotube	AMP	Co-TNT/GCE	SARS-CoV-2	NS	0.7 nM	14–1400 nM	[81]
Nanoparticle	CV	CeO_2_–TiO_2_/ITO	Uric acid	0.9016 μA·cm^−2^·mM^−1^	0.165 mM	10–700 mg·dL^−1^	[82]
Nanotube	CV	Cu_2_ONPs/TNT	Cholesterol	6034.04 µA·mM^−1^·cm^−2^	0.05 µM	24.4–622 µM	[83]
Nanoparticle	CV	DNA-TiO_2/_SPGE	H1N1 swine flu virus	40.32 μA·ng^−1^·cm^−2^	0.00024 ng·6 µL^−1^	0.0002–20 ng·6 µL^−1^	[84]
Nanoparticle	CV	Pt/CeO_2_-TiO_2_/LOx/Nf	Lactate	0.085 µA·µM^−1^·cm^−2^	5.9 µM	0.02–0.6 mM	[146]
Nanoparticle	CV	TiO_2_-CH/GCE	SARS-CoV-2 antibody	NS	3.42 ag·mL^−1^	50 ag mL^−1^–1 ng mL^−1^	[147]
Nanotube	CV	AgNPs/TiO_2_/TNT	Heat Shock Protein 70	NS	0.48 ng·mL^−1^	0.1–100 ng·mL^−1^	[148]
Nanoparticle	DPV	RGO@TiO_2_/ITO	Epithelial cell adhesion molecules	3.24 μA·mL ·ng^−1^·cm^−2^	6.5 pg·mL^−1^	0.01–60 ng·mL^−1^	[149]
Nanoparticle	CV and EIS	Mn-TiO_2/_SPE	Myoglobin	100.40 µA-cm^−2^.nM^−1^	0.013 nM	3–15 nM	[150]
Iron (II, III) Oxide-Based Electrochemical Biosensors							
Nanoparticle	DPV	Fe_3_O_4/_CeO_2_ @Au/GCE	MicroRNA-21	NS	0.33 fM	1 fM–1 nM	[93]
Nanoparticle	DPV	MWCNTs/Fe_3_O_4_/GCE	PSA	NS	0.39 pg·mL^−1^	2.5 pg·mL^−1^–100 ng·mL^−1^	[96]
Nanoparticle	DPV	GO/Fe_3_O_4_/PB	HBsAg	0.5 pg·mL^−1^ to 200 ng.mL^−1^	0.166 pg·mL^−1^	NS	[99]
Nanoparticle	DPV	Fe_3_O_4_-AuNPs-SPCE	HPV	2.4 μA·nM	0.1 nM	10^−4^ nM–1 μM	[100]
Nanoparticle	DPV	CS/Fe_3_O_4_/PPy/GCE	AβO	NS	3.4 fM	10 fM–10 μM	[101]
Nanoparticle	DPV	ds-DNA/p(L-Cys)/Fe_3_O_4_ NPs-GO/CPE	Adenine	NS	3.90 nM	0.01–30.0 μM	[102]
			Guanine		1.58 nM	0.01–25.0 μM	
Nanoparticle	DPV	Fe_2_O_3_–CH-S-Gr/AuE	Tannic acid	0.2 µA × µM^−1^	3.6 × 10^−3^ µM	0.01–1000 µM	[105]
Nanosheet	CV	GCE/Fe_3_O_4_/Gr-INPs/AChE	ATCh	255.6 μM·mM^−1^·cm^−2^	8.35 μM	12.5–112.5 μM	[151]
		GCE/Fe_3_O_4_/Gr-INPs/GOx	Glucose	700 μM·mM^−1^·cm^−2^	8.2 μM	12.5–112.5 μM	
Nanoparticle	DPV	Mag-Fe_3_O_4/_GCE	Uric acid	NS	7.5 μM	7.5 μM–0.18 mM	[152]
Nanoparticle	CV	GOx/PVA-Fe_3_O_4_/Sn	Glucose	9.36 mA·mM^−1^	8 mM	5 × 10^−3^–30 mM	[153]
Nickel (II) Oxide-Based Electrochemical Biosensors							
Nanoparticle	DMM	CH/NiO/ITO	AChE	58 mV·decade^−1^	NS	0.01–0.2 mM	[111]
Nanodentrites	CA	Au@NiO NDMA	Glucose	11.46 μA·μM^−1^·cm^−2^	0.1 μM	10–5000 μM	[116]
			Lactate	11.89 μA·μM^−1^·cm^−2^	8.2 μM	100–10,000 μM	
Nanoparticle	DPV	NiONPs/MXen/GCE	Methamphetamine	NS	333.3 fM	10 pM–50 mM	[119]
Hollow sphere	CV	3D-NiO hollow sphere/RGO/GCE	Glucose	2.04 mA·mM^−1^·cm^−2^	82 nM	0.009–1.129 mM	[154]
Nanoflake	CV	NiO/Mn_2_O_3_	Glucose	167 μA·mM^−1^·cm^−2^	NS	10–2860 μM	[155]
Copper (II) Oxide-Based Electrochemical Biosensors							
Nanoflower	CA	NFS-CuO/Ag/SiNPs/GCE	Glucose	4877.6 μA·mM^−1^·cm^−2^	0.1 μM	0.1 μM–2.5 μM	[122]
Nanoleaves	CV	CuO/GCE	Glucose	1467.32 μA·mM^−1^·cm^−2^	12 nM	0.005–5.89 mM	[124]
Nanoparticle	DPV	ZnO-CuO/SPE	*E. coli*	11.04 μA·CFU^−1^	2 CFU·mL^−1^	1 × 10^3^–8 × 10^4^ CFU·mL^−1^	[125]
Nanoparticle	DPV	CuO/WO_3_-GO	Cancer cells	NS	18 cells·mL^−1^	50–10^5^ cells·mL^−1^	[126]
Nanoparticle	CV	CuO-MgO/GCE	Dopamine	69 μA·cm^−2^·mM^−1^	6.4 μM	10–100 μM	[127]
Nanoparticle	DPV	CH/CuO NPs/ERGO/GCE	HAS	NS	10–450 ng·mL^−1^	2.6 ng·mL^−1^	[130]
Nanoparticle	CV	SB3C16@Cu_2_O/SPCE	Creatinine	NS	5.0 μM	10–200 μM	[133]
Nanoparticle	SWV	CuONPs/PGE	Testosterone	NS	4.6 nM	5–200 nM	[134]
Nanoparticle	DPV	Tyr/Cu_2_O NPs/GCE	Dopamine	NS	0.3 μM	10–70 µM	[135]
Nanoparticle	CV	Mn_3_O_4_/NiO/CNFs/GCE	Glucose	243.74 µA·mM^−1^·cm^−2^	0.73 µM	3000–12,000 µM	[136]
Nanoparticle	DPV	Pt–CuO/CP	H_2_S	NS	0.5 ppm	0.5–500 ppm	[156]
Other Nanostructured Metal Oxide-Based Electrochemical Biosensors							
Nanorod	EIS	CeO_2_-NR@Ppy/CP	*Salmonella*	593.7 Ω·nM ^−1^·cm^−2^	0.084 nM	0.01–0.4 nM	[137]
Nanosheet	CV	BSA/anti-SAA/APTES/nZrO_2_@PC/ITO	Serum Amyloid A	95.88 μA [log(μg mL^−1^)]^−1^ cm^−2^	6.37 μg·mL^−1^	10–100 μg·mL^−1^	[138]
Nanoparticle	CV	Cu/SnO_2_/ITO	Lung cancer biomarkers	NS	1 ppb	20–100 ppb	[139]
		Mn/SnO_2_/ITO			0.1 ppb	1–1000 ppb	
Nanorod	EIS	APTES/WO_3_ NRs/ITO	Troponin I	6.81 [KΩ.mL/(ng·cm^2^)]	0.01 ng·mL^−1^	0.01–10 ng·mL^−1^	[140]
Nanoparticle	DPV	Nf/GOx/MgO/ENIG/Cu/PI	Glucose	17.45 mV·mM^−1^	0.11 mM	2–10 mM	[141]
Nanofiber	AMP	Al/β-Bi_2_O_3_	Serotonin	51.64 μA·nM^−1^	0.29 nM	10 nM–1 μM	[157]
Nanoparticle	CV	AChE-Nf/MnCo_2_O_4.5_HoQS-MPs/GCE	Monocrotophos	NS	1.82 × 10^−14^ M	0.1 pM–100 nM	[158]
			Methamidophos		1.66 × 10^−14^ M	0.1 pM–10 nM	
			Carbaryl		1.58 × 10^−14^ M	0.1 pM–10 nM	

AChE: acetylcholine esterase; AMP: amperometry; APTES: 3-aminopropyltrie thoxysilane; AT: air-annealed; ATCh: acetylthiocholine; Au: gold; AβO: amyloid-beta oligomers; BSA: bovine serum albumin; CA: chronoamperometry; CS: chondroitin sulfate; CH: chitosan; CNFs: carbon nanofibers: Co: co-functionalized; CP: carbon paper; CPE: carbon paste electrode; CV: cyclic voltammetry; Cys: cysteine; DMM: digital multimeter; DPV: differential pulse voltammetry; EIS: electrochemical impedance spectroscopy; ENIG: electroless nickel immersion gold; ERGO: electrochemically reduced graphene oxide; FTO: fluorine-doped tin oxide; GCE: glassy carbon electrode; GE: graphite electrode; GO: graphene oxide; GOx: glucose oxidase; Gr: graphite; Hb: hemoglobin; HPV: human papillomavirus; HoQS: hollow quadruple-shelled porous; HSA: human serum albumin; IgG: immunoglobulin G; INPs: iron-based nanoparticles; ITO: indium tin oxide; LOD: limit of detection; LOx: lactate oxidase; Mag: Magneto; MPs: micropolyhedrons; MWCNTs: multi-walled carbon nanotubes; NDMA: nanodentrite microarrays; Nf: Nafion; NFS: nanoflower shaped; NPs: nanoparticles; NWs: nanowires; NS: not stated; PB: Prussian blue; PC: mesoporous carbon nanosheets; PI: polyimide; PPy: polypyrrole; PSA: prostate-specific antigen; PT: plasma treatment; PVA: polyvinyl alcohol; QDs: quantum dots; RGO: reduced graphene oxide; RT: response time; SB3C16: sulfonate-based zwitterion; SAA: Serum Amyloid A; SPCE: screen-printed carbon electrode; SPE: screen-printed electrode; SPGE: screen-printed gold electrode; SSZ: sulfasalazine; S-Gr: sulfur-doped graphene substrate; SWV: square wave voltammetry; TNT: titanium dioxide nanotubes; TPIPs: thermal responsive protein imprinted polymers; Tyr: tyrosinase; Urs: urease enzyme.
